# Repair of large traumatic tympanic membrane perforation using ofloxacin otic solution and gelatin sponge

**DOI:** 10.1016/j.bjorl.2020.03.007

**Published:** 2020-05-05

**Authors:** Xiuguo Li, Hui Zhang, Yuanyuan Zhang

**Affiliations:** a Jining NO.1 People's Hospital, Department of Otolaryngology, Jining, China; b Jining Medical University, Department of Histology and Embryology, Jining, China

**Keywords:** Traumatic, Tympanic membrane perforation, Gelatin sponge, Ofloxacin otic solution, Spontaneous healing

## Abstract

**Introduction:**

Traumatic large tympanic membrane perforations usually fail to heal and require longer healing times. Few studies have compared the healing and hearing outcomes between gelatin sponge patching and ofloxacin otic solution.

**Objectives:**

To compare the healing outcomes of large traumatic tympanic membrane perforations treated with gelatin sponge, ofloxacin otic solution, and spontaneous healing.

**Methods:**

Traumatic tympanic membrane perforations >50% of the entire eardrum were randomly divided into three groups: ofloxacin otic solution, gelatin sponge patch and spontaneous healing groups. The healing outcome and hearing gain were compared between the three groups at 6 months.

**Results:**

A total of 136 patients with large traumatic tympanic membrane perforations were included in analyses. The closure rates were 97.6% (40/41), 87.2% (41/47), and 79.2% (38/48) in the ofloxacin otic solution, gelatin sponge patch, and spontaneous healing groups, respectively (*p* = 0.041). The mean times to closure were 13.12 ± 4.61, 16.47 ± 6.24, and 49.51 ± 18.22 days in these groups, respectively (*p* < 0.001).

**Conclusions:**

Gelatin sponge patch and ofloxacin otic solution may serve as effective and inexpensive treatment strategies for traumatic large tympanic membrane perforations. However, ofloxacin otic solution must be self-applied daily to keep the perforation edge moist, while gelatin sponge patching requires periodic removal and re-patching.

## Introduction

Traumatic Tympanic Membrane Perforations (TMPs) tend to heal spontaneously, although large perforations may fail to heal and require longer healing times.^1^, ^2^ Various biological materials have been applied as patches to act as scaffolds for epithelial migration into the eardrum and facilitate eardrum healing to reduce the healing time and improve the closure rate of large TMPs.^3^, ^4^, ^5^, ^6^ Gelatin sponge is a commonly used biological material which has been used as a support or for packing in middle ear surgery, and has also been shown to be effective for the repair of small chronic TMPs and large traumatic TMPs.^7^, ^8^, ^9^ In addition, ofloxacin otic solution is a broad-spectrum quinolone antibiotic widely used to treat acute and chronic otitis externa, suppurative otitis media, and for myringoplasty.^10^, ^11^

Recent clinical and experimental studies have demonstrated that topical application of ofloxacin otic solution accelerates the healing of large traumatic TMPs.^12^, ^13^, ^14^, ^15^ However, few studies have compared the healing and hearing outcomes of large traumatic TMPs between gelatin sponge patching and ofloxacin otic solution. This study compared the healing and hearing outcomes of gelatin sponge patching, ofloxacin otic solution, and spontaneous healing in cases of large traumatic TMPs.

## Methods

### The statement of approval of the institution's ethics committee

The study protocol was approved by the Human Research Ethics Committee of Jining NO.1 People's Hospital, Shandong Province, China under n° 20130912, which is guided by local policy, national laws, and the World Medical Association Declaration of Helsinki. Informed consent was obtained from all participants.

### Materials

Subjects were recruited consecutively from patients diagnosed with traumatic TMPs who visited the Department of Otolaryngology of our hospital between January 2014 and December 2018. The inclusion criteria were as follows: traumatic TMP, age >14 years, perforation of at least 50% of the pars tensa, and duration of perforation less than 1 week. The exclusion criteria were as follows: preexisting myringosclerosis, middle ear infection or severe vertigo at the time of the hospital visit, suspicion of ossicular chain damage or granulation tissue hyperplasia, traumatic perforations resulting from electrowelding or chemical injury, and history of middle ear disease in the ipsilateral or contralateral ear.

Ages, sex, duration of injury, cause of injury, position, size of the TMP and presence or absence of otorrhea were recorded at the time of each hospital visit. Each patient was examined endoscopically after removal of cerumen and/or blood clots in the external auditory canal (EAC) with a cotton bud soaked in povidone-iodine solution, and the site and size of the perforation were documented. Standard pure-tone audiometric testing was performed at the initial and final visits after treatment. Pure-tone averages were determined for air and bone conduction at 500, 1000, 2000, and 4000 Hz. The sizes of TMPs were analyzed with Image J software (National Institutes of Health, Bethesda, MD), and perforation size was graded into large and subtotal perforation (50% and 75% of the eardrum, respectively).

The principal investigator, aided by a registered nurse, allocated patients to various treatments with simple random sampling. Specifically, consecutive subjects who fulfilled the inclusion criteria and signed the consent form were assigned random numbers generated by SPSS 19.0 for Windows (IBM, Chicago, IL), which allocated them to one of three groups: ofloxacin otic solution (*n* = 50), gelatin sponge patch (*n* = 50), and spontaneous healing (*n* = 50).

## Technical methods

### Ofloxacin otic solution group

The EAC was cleaned with a cotton bud soaked in povidone-iodine solution. The perforation edges were not approximated and no scaffolding material was applied. Approximately, 0.2–0.3 mL (2–3 drops) of ofloxacin otic solution (WanHe, ShenZhen City, China) was self-applied to the remnant tympanic membrane twice daily by the patient; the perforated ear was angled upward for at least 30 min following application to ensure that the remnant tympanic membrane remained moist.

### Gelatin sponge patch group

The EAC was cleaned with a cotton bud soaked in povidone-iodine solution. The edges of the perforation were not approximated. A modified compressed gelatin sponge sheet, larger than the perforation, was soaked in 0.5% (w/v) erythromycin ointment and placed onto the tympanic membrane remnant (i.e., an onlay technique) to completely cover the perforated area with margins of at least 2 mm.

### Spontaneous healing group

The ear was kept dry and no intervention was provided, but all patients underwent regular follow-up.

### Follow-up

The first follow-up was scheduled for 3 days after initiation of treatment in the ofloxacin otic solution group to confirm that the patients had self-applied the drops correctly, and eardrop dosage was adjusted carefully to ensure that the surface of the eardrum remained moist (i.e., neither dry nor overly wet). Any inappropriate patient technique was corrected. Thereafter, follow-up was scheduled twice a week within 1 month after treatment, and subsequently once a week until complete closure of the perforation was achieved or for up to 6 months. Patients were advised to reduce the number of ear drops and to take oral amoxicillin (with or without application of ofloxacin drops) if purulent otorrhea developed.

In the gelatin sponge group, the sponge was removed, and a fresh piece of sponge was placed onto the tympanic membrane at each visit. The tympanic membrane was repeatedly examined endoscopically, and color photographs were obtained by an independent clinician blinded to group allocation at all follow-up visits.

Infection was defined as the presence of purulent otorrhea with otalgia in the EAC or middle ear on clinician examination. The presence of limpid water otorrhea was not classified as an infection. The closure rate, mean time to closure, hearing gain, and infection rate were evaluated at 6 months.

### Statistical methods

Results are given as means ± standard deviation or as percentages. For statistical analyses of the results of the three groups, one-way analysis of variance or Kruskal–Wallis test was used for continuous data, and the *χ*^2^ test was used for categorical data. A two-sample *t* test or Mann–Whitney *U* test was used to compare the results of two groups. The rate of otorrhea was compared between the ofloxacin otic solution, gelatin sponge patch, and observation groups by Fisher's exact test. In post hoc multiple comparisons, *p* = 0.0167 (0.05/3) was considered indicative of a significant difference. Otherwise, a value of *p* < 0.05 was taken to indicate statistical significance. Statistical analyses were performed using SPSS software (version 19.0 for Windows; IBM).

## Results

### Demographic characteristics

**Figure 1:**
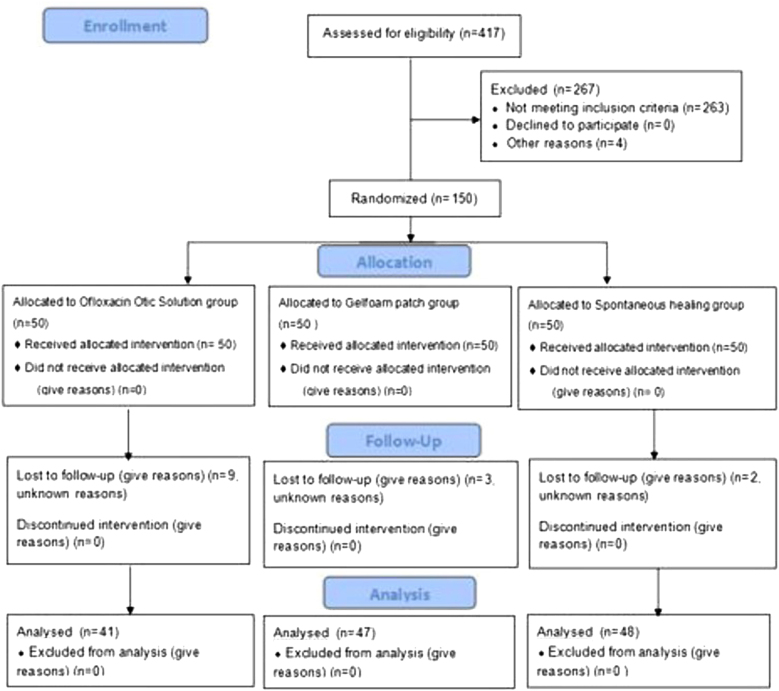
CONSORT 2010 flow diagram.

A total of 150 patients were randomized into this study. However, only 136 patients with a diagnosis of large traumatic TMP were included in the final analysis. Nine patients in the ofloxacin otic solution group, three patients in the gelatin sponge patch group, and two patients in the spontaneous healing group were lost to follow-up (Fig. 1). Demographic data are shown in Table 1. The sex, age, side of the ear, cause of the injury, position and size of the perforation, and duration of injury were matched among the three groups (*p* = 0.84).

**Table 1:** Demographic data of ofloxacin otic solution, gelfoam patching and spontaneous healing groups.

	Ofloxacin otic solution group	Gelfoam patching group	Spontaneous healing group	*p*-Value
N°	41	47	48	–
Sex (M/F)	14/27	16/31	19/29	0.399^a^
Side (R/L)	9/32	10/37	9/39	0.093^a^
Age, year	37.04 ± 11.12	38.53 ± 10.27	36.57 ± 12.41	0.429^b^
Position (A/P/KS)	18/3/20	22/4/21	25/3/20	0.632^a^
Cause of perforation (S/B)	39/2	44/3	46/2	0.279^a^
Duration, day	3.24 ± 1.13	3.16 ± 2.56	3.17 ± 2.46	0.187^b^
Hearing level, dB	29.7 ± 6.0	30.4 ± 5.3	31.1 ± 6.2	0.974^b^

Continuous variables was expressed using mean ± SD, categorical variables were expressed using *n* or *n* (%).

a Chi-square test/*χ*^2^ test.

b One-way analysis of variance, ANOVA. S, slap injury; B, blast injury; A, anterior; P, posterior; KS, kidney-shaped.

### Healing outcomes

The healing outcomes are shown in Table 2. The closure rates were 97.6% (40/41), 87.2% (41/47), and 79.2% (38/48) in the ofloxacin otic solution, gelatin sponge patch, and spontaneous healing groups, respectively (*p* = 0.041). The closure rate differed significantly between the ofloxacin otic solution and spontaneous healing groups (*p* = 0.008) but not between the other two pairings (ofloxacin otic solution vs. gelatin sponge patch, *p* = 0.037; gelatin sponge patch vs. spontaneous healing, *p* = 0.642).

**Table 2:** The healing outcome of ofloxacin otic solution, gelfoam patching, and spontaneous healing groups.

	Closure rate (%)	P^2,^^a^	Mean closure time (days)	P^2,^^b^	Hearing gain, dB
Ofloxacin otic solution group (*n* = 41)	97.6	0.037^a^	13.12 ± 4.61	0.069^a^	13.0 ± 1.5
Gelfoam patching group (*n* = 47)	87.2	0.642^b^	16.47 ± 6.24	0.001^a^	12.8 ± 3.1
Spontaneous healing group (*n* = 48)	79.2	0.008^c^	49.51 ± 18.22	0.001^c^	12.7 ± 2.6

P^1^	0.041		0.001		0.79

P^1^, Comparison between three groups in terms of closure rate, or mean closure time, or Hearing gain.

P^2^, Comparison between two by two in terms of closure rate, or mean closure time.

a Comparison between ofloxacin otic solution and gelfoam patching groups.

b Comparison between gelfoam patching and spontaneous healing groups.

c Comparison between ofloxacin otic solution and spontaneous healing groups.

The mean times to closure were 13.12 ± 4.61, 16.47 ± 6.24, and 49.51 ± 18.22 days in the ofloxacin otic solution, gelatin sponge patch, and spontaneous healing groups, respectively (*p* < 0.001). The mean times to closure differed significantly between the ofloxacin otic solution and spontaneous healing groups (*p* < 0.001) and between the gelatin sponge patch and spontaneous healing groups (*p* < 0.001) but not between the ofloxacin otic solution and gelatin sponge patch groups (*p* = 0.069).

**Figure 2:**
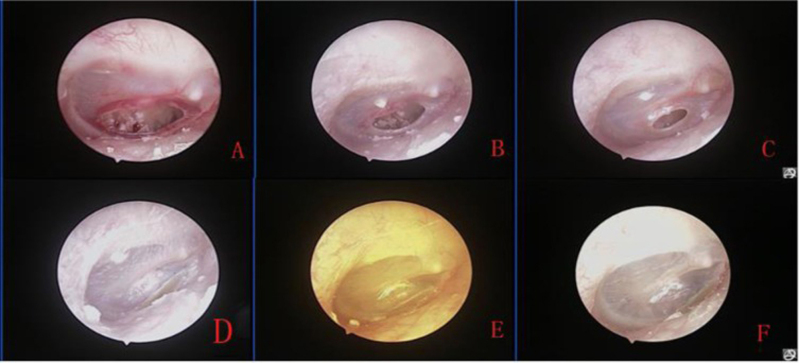
The healing process of large perforation after topical application of ofloxacin otic solution: 1st after perforation (A), and 4 days (B), 7 days after treatment (C), and 2 weeks (D), 3 weeks (E) and 7 weeks after treatment (F).

Fig. 2 shows the healing process of the patients with traumatic TMP treated with ofloxacin otic solution.

### Hearing outcome and complications

The mean hearing improvements at final visits after treatment were 13.0 ± 1.5 dB for the ofloxacin otic solution group, 12.8 ± 3.1 dB for the gelatin sponge patch group, and 12.7 ± 2.6 dB for the spontaneous healing group. Differences in hearing improvement rates among the three groups were not statistically significant (*p* = 0.79).

Otorrhea was observed in 26/41 (63.4%) patients in the ofloxacin otic solution group, 6/47 (12.8%) patients in the gelatin sponge patch group, and 3/48 (6.25%) patients in the spontaneous healing group. However, defined infection and purulent otorrhea were only seen in three patients in the spontaneous healing group. No treatment-related complications, such as otomycosis, severe vertigo, or external auditory meatus hyperkeratosis were observed in any of the treatment groups. Three patients complained of ear pain in the spontaneous healing group. Only 19 patients reported ear discomfort due to the presence of otorrhea in the ofloxacin otic solution group. In addition, endoscopic examination confirmed that the TMP had been closed once the patients reported that the symptoms of tinnitus and aural fullness had disappeared in the ofloxacin otic solution group.

## Discussion

Although traumatic TMP is a common entity in otology clinics, there is debate regarding its optimal treatment. Some groups prefer spontaneous healing because of the high rate of spontaneous healing in cases of traumatic TMP. However, large traumatic TMPs have a lower rate of spontaneous healing and require longer healing times. Lou et al.^2^ reported a closure rate of 80% in 20 large perforations over a 12 month follow-up period. In long-term observation of spontaneous closure of traumatic tympanic membrane perforation, Tachibana et al.^16^ reported a spontaneous healing rate of 67.5% (27/40); the time to closure was more than 3 months in 7 of these 27 patients. Others have suggested that biological material patching combined with edge approximation may reduce the healing time and improve the time to closure of large traumatic TMPs.^3^, ^4^, ^5^, ^6^ However, recent studies have shown that edge approximation may not be necessary, and that single patching can obtain similar results.^3^, ^7^ The previous consensus view was to keep the EAC dry and avoid use of antibiotic ear drops.^1^, ^16^, ^17^ By contrast, some recent studies have shown that topical application of ear drops reduces healing time and improves the time to closure of large traumatic TMPs.^14^, ^15^, ^18^

Some biological materials have not been widely used clinically because of expense and inconvenience.^3^, ^4^, ^5^, ^6^ While the adoption of growth factor solutions has been restricted around the world because of cost and safety concerns, ofloxacin otic solution and gelatin sponge are inexpensive, safe, and have been widely used in otology surgery and for the treatment of inflammatory diseases. Recent clinical and experimental studies have suggested that gelatin sponge and ofloxacin otic solution aid eardrum healing.^7^, ^8^, ^14^, ^15^

However, it remains unclear which treatment is more suitable for application in cases of traumatic TMP. This study demonstrated that gelatin sponge patches and ofloxacin otic solution significantly reduced the time to closure of large TMPs compared to spontaneous healing. The closure rate in the ofloxacin otic solution group was significantly higher than that of the spontaneous healing group. The closure rate was also higher in the ofloxacin otic solution group than the gelatin sponge patch group (97.6% vs. 87.2%), although the difference was not significant. In addition, the improvement in healing was not statistically significant among the three groups. Systemic antibiotics were not applied in this study, although the rate of otorrhea was higher in the ofloxacin otic solution group, defined as infection and purulent otorrhea, which was only seen in three patients in the spontaneous healing group. This may have been because additional erythromycin ointment had a topical anti-inflammatory effect in the gelatin sponge patch group, while ofloxacin otic solution itself was an antibiotic solution.

The times to closure of large TMPs were significantly reduced in the gelatin sponge patch and ofloxacin otic solution groups compared to the spontaneous healing group. Although the healing time was not significantly different between the gelatin sponge patch and ofloxacin otic solution groups, the mean healing time in the gelatin sponge patch group was delayed by 3 days compared to the ofloxacin otic solution group. Gelatin sponge patching was performed by the physician, did not require replacing the patch every day, and did not place any technical demands on the patients. The gelatin sponge patch was replaced regularly based on the objectives of this study; however, removing the gelatin sponge slightly injured the healing eardrum because of the added erythromycin ointment at each follow-up visit. However, frequent replacement of the gelatin sponge would not be needed in clinical treatment. Use of the gelatin sponge patch was suitable for patients failing to attend timely follow-ups or those living far away for the clinic. By contrast, ofloxacin otic solution was self-applied daily by the patients to keep the eardrum moist. Lou et al.^14^, ^15^ reported that the best method was to keep the perforation edges moist and avoid excessive dry and immersion wetting. Excessive application would result in otorrhea and cause discomfort of the EAC. However, keeping the perforation edges moist is a technical challenge for patients, and therefore follow-up a few days before each application is very important to correct the number of eardrops and application method. In addition, in our study, the application of ofloxacin otic solution was affected by the structure of the EAC, the position of perforation, and eustachian tube function. More ofloxacin otic solution would remain at the EAC and result in a greater requirement for solution in patients with tortuous EAC; the upper perforations required more solution to moisten the perforation edges compared to lower perforations of the eardrum. Similarly, patients with unblocked eustachian tube also required more applications of solution because some solution entered the nasopharynx. Therefore, it is impossible to determine the optimal amount of ofloxacin otic solution to apply. Nevertheless, treatment with ofloxacin otic solution could be fitted for the patients with timely follow-up. In this study, the TMP was confirmed to be closed by endoscopy once the patients reported the disappearance of symptoms of tinnitus and aural fullness in the ofloxacin otic solution group. This could have resulted in the higher rate of loss to follow-up. However, although the patients reported the disappearance or improvement of symptoms, remnant perforations were seen by endoscopy after removing the gelatin sponge in a few patients, because gelatin sponge patch provisionally closed the perforations and thereby resulted in improvement of symptoms. Thus, the application of ofloxacin otic solution may be stopped by patients once the symptoms have disappeared, although the disappearance of the symptoms did not necessarily indicate closure of the TMPs during the process of follow-up in the gelatin sponge patch group.

The limitation of this study was that the number of days of healing could not be objectively evaluated because of the follow-up schedule of twice a week for 1 month and subsequently once a week after treatment.

## Conclusion

Gelatin sponge patch and ofloxacin otic solution significantly reduced the time to closure of large TMPs compared to spontaneous healing and did not affect hearing improvement or rate of middle ear infection. Thus, these two treatments may represent viable strategies for large traumatic TMPs because they are inexpensive and can be obtained easily. However, the ofloxacin otic solution group had a higher closure rate and shorter time to closure compared to the gelatin sponge patch group, although it had to be self-applied daily to keep the perforation edge moist and so had high technical requirements for patients. By contrast, the removal of the gelatin sponge or re-patching was needed after sponge patch treatment.

## Conflicts of interest

The authors declare no conflicts of interest.
